# Supplementary material for: Nutlin-3a induces KRAS mutant/p53 wild type lung cancer specific methuosis-like cell death that is dependent on GFPT2
Source: J Exp Clin Cancer Res. 2023 Dec 14;42:338. doi: 10.1186/s13046-023-02922-8 (PMC10720203; doi:10.1186/s13046-023-02922-8)
Supplement: Supplementary file 1 — Additional file 1. [file 13046_2023_2922_MOESM1_ESM.docx]

**Supplementary Information**

**Nutlin-3a induces *KRAS* mutant/*p53* wild type lung cancer specific methuosis-like cell death that is dependent on GFPT2**

Dasom Kim^1,2^, Dongwha Min^1,2^, Joohee Kim^3^, Min Jung Kim^3^, Yerim Seo^4^, Byung Hwa Jung^4,5^, Seung‑Hae Kwon^6^, Hyunju Ro^7^, Seoee Lee^7^, Jason K. Sa^8^, Ji-Yun Lee^1^*

^1^Department of Pathology, College of Medicine, Korea University, Seoul, South Korea

^2^Department of Biomedical Science, College of Medicine, Korea University, Seoul, South Korea

^3^ Department of Biological Science, Sookmyung Women’s University, Seoul, South Korea

^4^ Center for Advanced Biomolecular Recognition, Korea Institute of Science and Technology (KIST), Seoul 02792, Korea

^5^Division of Bio-Medical Science and Technology, KIST School, Korea University of Science and Technology (UST), Seoul, 02792, South Korea

^6^ Korea Basic Science Institute, Seoul Center, Seoul, South Korea

^7^Department of Biological Sciences, College of Bioscience and Biotechnology, Chungnam National University, Daejeon 34134, Korea

^8^Department of Biomedical Sciences, Korea University College of Medicine, Seoul, South Korea

**Correspondence to:* Ji-Yun Lee, Ph.D., Department of Pathology, Korea University College of Medicine, 73, Goryeodae-ro, Seongbuk-gu, Seoul 02841, South Korea

Tel: +82 22906141; Fax: +82 29533130; E-mail: [jiyun-lee@korea.ac.kr](mailto:jiyun-lee@korea.ac.kr)

**Supplementary Figures**

**
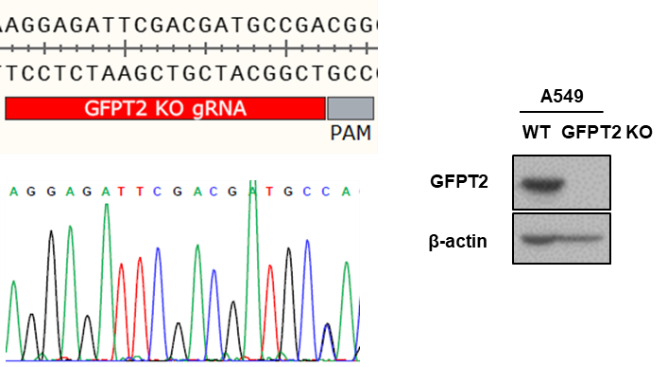
**

**Suppl. Fig. 1 Generation of GFPT2 knockout A549 cell by CRISPR-Cas9.** Knockout of GFPT2 was verified by genomic sequencing and western blotting.

**
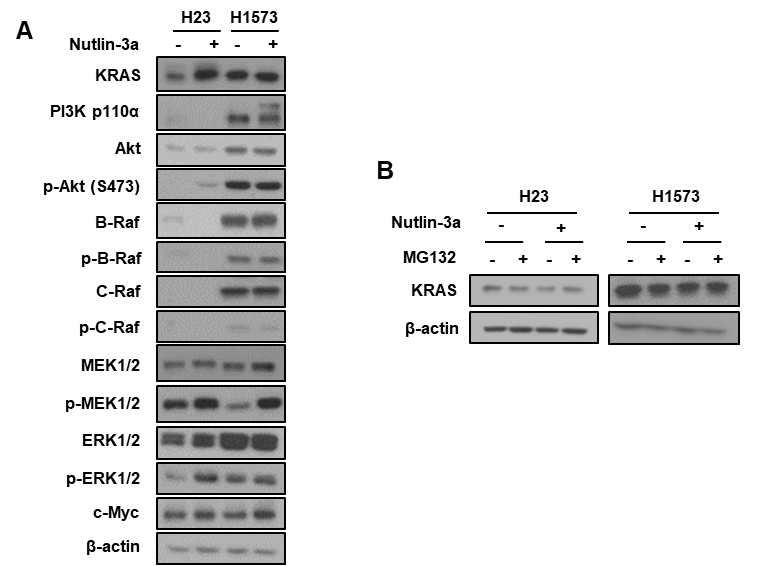
**

**Suppl. Fig. 2 Nutlin-3a did not affect KRAS, and KRAS signaling in *KRAS* MT/*p53* MT NSCLC cells.** Cells were treated with nutlin-3a (30 μM) for 24h. **A** Expression of KRAS, and KRAS down signaling molecules were examined by western blotting. **B** KRAS stability was examined by western blotting after treatment of MG132 (10 μM) for 6h before harvest.

**
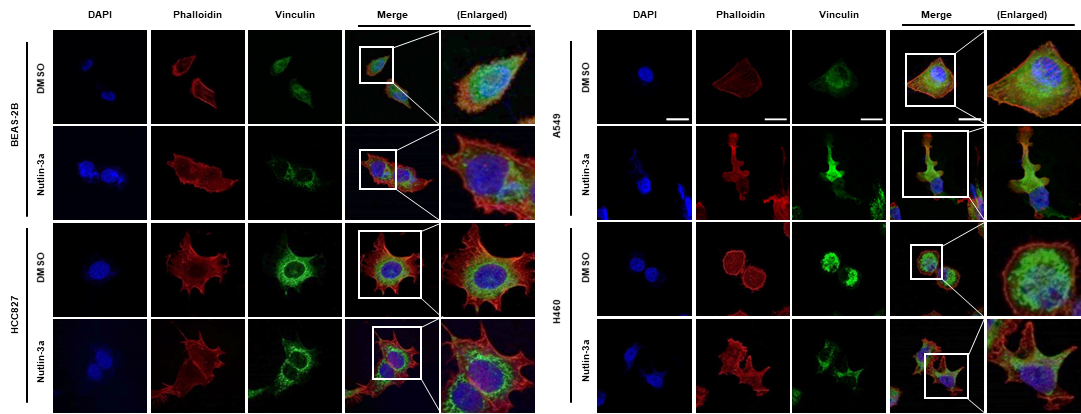
**

**Suppl. Fig. 3** **The cytoskeleton is not affected upon treating *KRAS* wild type cells with nutlin-3a.** Cells were stained with rhodamine–phalloidin (red), vinculin (green) and nuclei were counterstained with DAPI (blue) after treatment with nutlin-3a (30 μM) for 24 h. Representative images obtained using confocal laser scanning microscopy are shown. Scale bar: 20 μm.

**
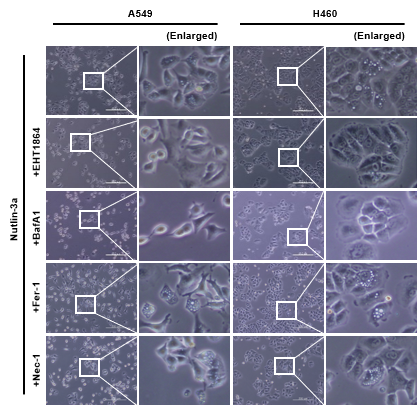
**

**Suppl. Fig. 4 Nutlin-3a-induced vacuolization is recovered by macropinocytosis inhibitors.** Cells were treated with nutlin-3a (30 μM) for 24 h in the absence or presence of EHT1864 (10 μM), bafilomycin A1 (BafA1) (5 nM), ferrostatin-1 (Fer-1) (1 μM), and necrostatin-1 (Nec-1) (10 μM). Representative images captured using light microscopy are shown. EHT1864 and bafilomycin A1, but not ferrostatin-1 or necrostatin-1, rescue nutlin-3a-induced vacuolization. Scale bar: 100 μm.


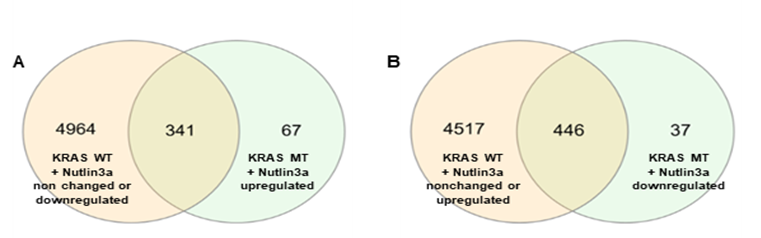

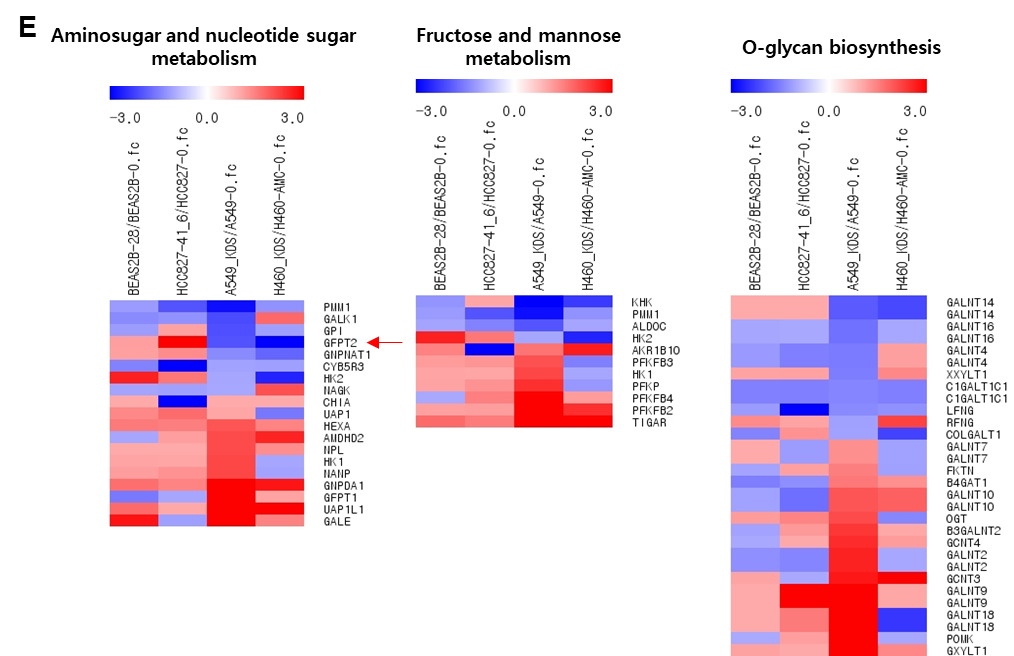

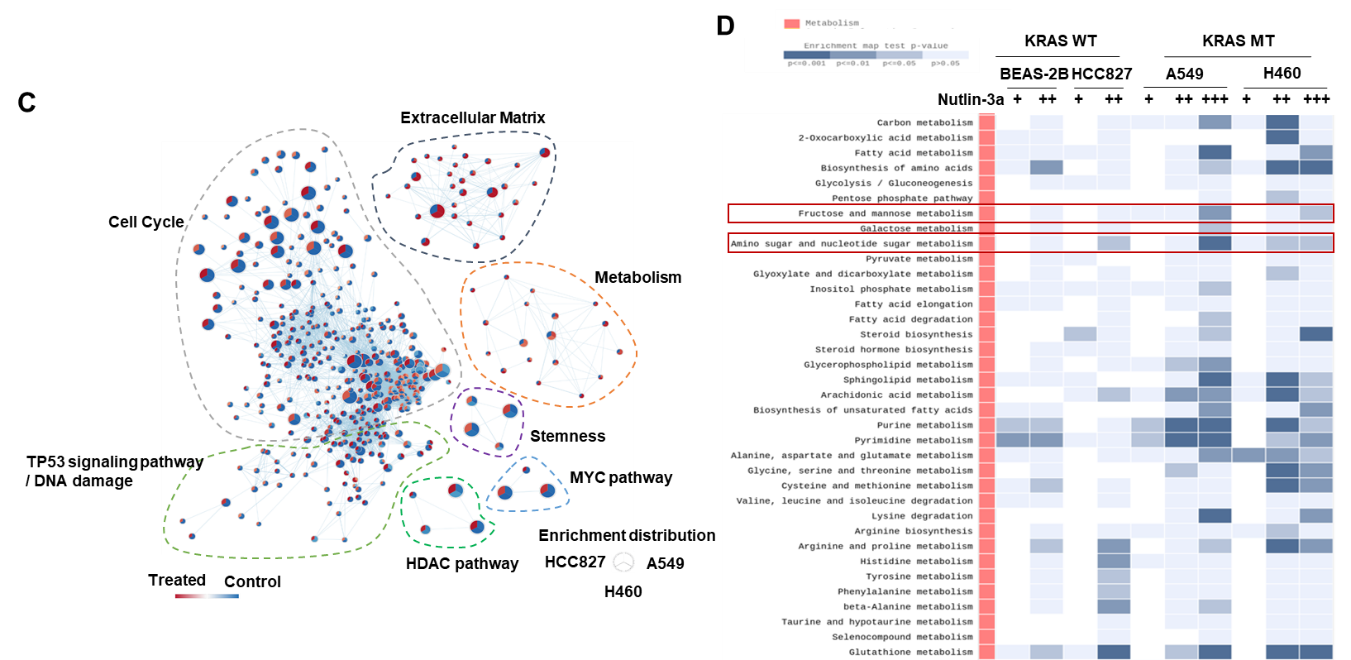


**Suppl. Fig. 5 Analysis of RNA sequencing results showed nutlin-3a affected in HBP related metabolic pathways in *KRAS* MT/*p53* WT cells.** **A, B** Venn diagrams of up- (A) and downregulated (B) genes in *KRAS* MT/*p53* WT NSCLC cells (A549, and H460) compared to those in *KRAS* WT cells (BEAS-2B, and HCC827) after nutlin-3a treatment. The list of overlapping genes is provided in Tables S2 and S3. **C, D** Reactom (C) and KEGG (D) pathway analyses showed the enrichment of metabolic pathway. **E** Nutlin-3a treatment changed HBP associated metabolic pathways in *KRAS* MT/*p53* WT NSCLC cells. Heatmap of DEGs from the ‘Amino sugar and nucleotide sugar metabolism’, ‘Fructose and mannose metabolism’, and ‘O-glycan biosynthesis’ between *KRAS* WT, and *KRAS* MT/*p53* WT (A549, and H460) and *KRAS* WT cells after treatment with a high concentration of nutlin-3a. Each of *KRAS* WT cells were treated with IC_50_, 24h concentration of nutlin-3a for 48 h, and *KRAS* MT/*p53* WT cells were treated with 30 μM of nutlin-3a for 24 h.

**
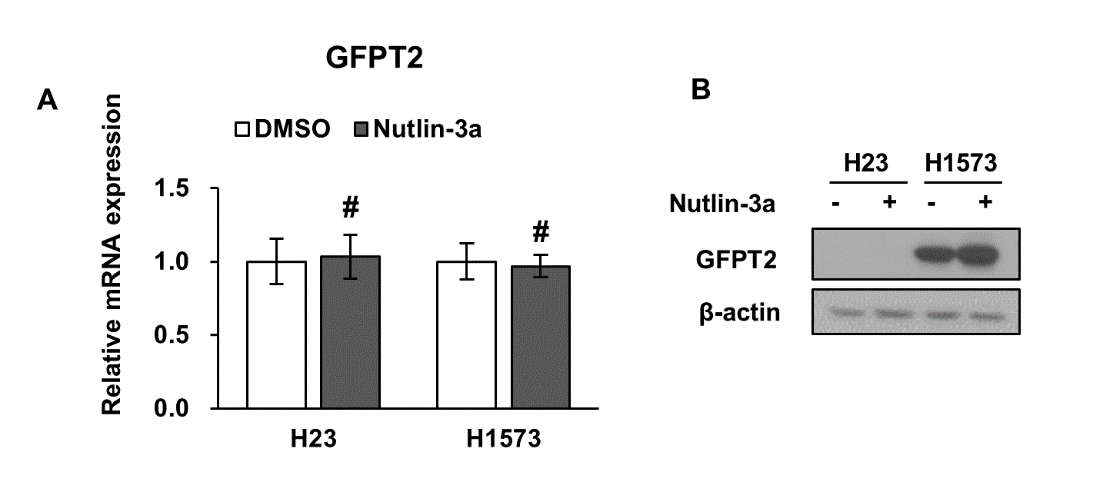
**

**Suppl. Fig. 6 Nutlin-3a did not decrease GFPT2 in *KRAS* MT/*p53* MT NSCLC cells.** Expression of *GFPT2* was examined by RT-qPCR (A), and western blotting (B) after cells were treated with nutlin-3a (30 μM) for 24h.


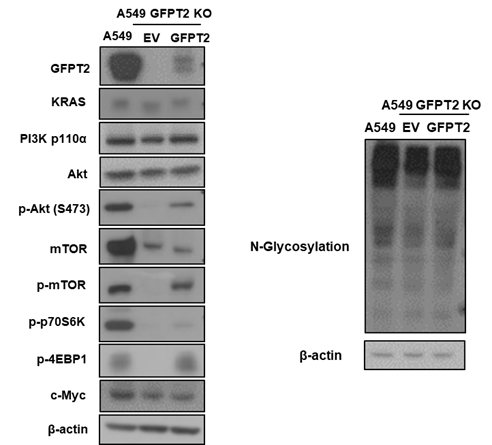


**Suppl. Fig. 7 GFPT2 regulated KRAS-PI3K/AKT-mTOR pathway, and N-glycosylation.** GFPT2 was re-expressed by transfection of GFPT2-HA for 24h into GFPT2 knockout A549 cells. GFPT KO cells showed decreased KRAS-AKT/mTOR signaling molecules as well as N-glycosylation, which was recovered by re-expression of GFPT2.

**
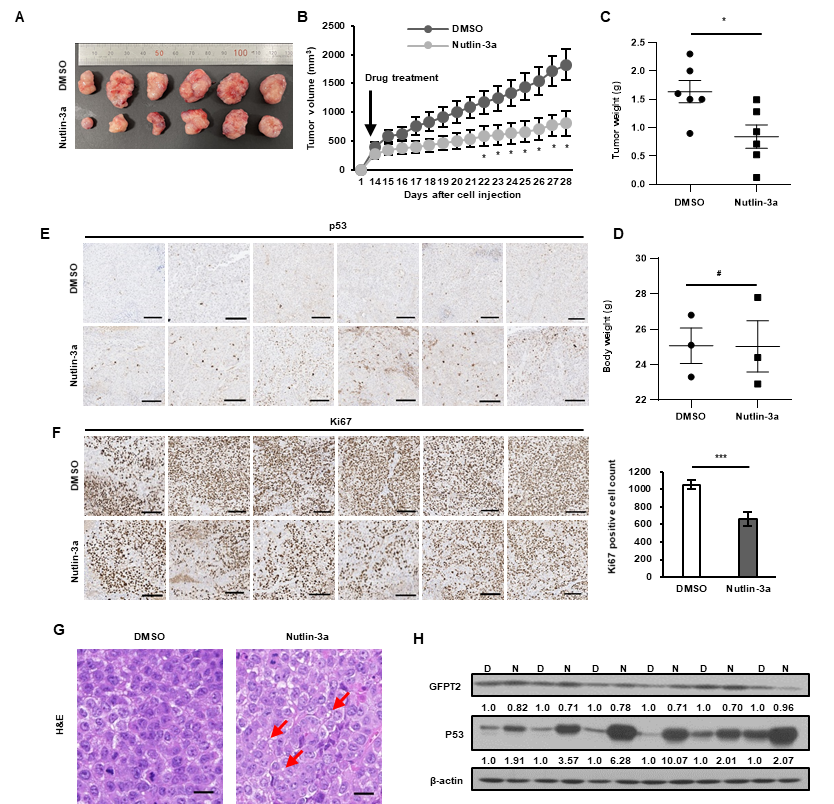
**

**Suppl. Fig. 8 Verification of nutlin-3a’s anticancer effects in H460 tumor xenograft model.** **A, B** Images (A) and growth rates (B) of subcutaneous tumors formed by H460 injection in the presence and absence of nutlin-3a. **C** Weight of the tumors shown in (A). **D** Weight of mice treated with either vehicle control or nutlin-3a on the day of euthanasia. **E-G** Immunohistochemical analysis of (E) p53 and (F) Ki67 expression and (G) H&E staining of tumor tissues. **H** Western blot analysis of GFPT2 and p53 expression in *in vivo* xenograft tumors. The densitometry quantification of the western blot determined using Image J software (Ver. 1/52n, NIH). Three images per tissue were used for Ki67 quantification (F), Red arrows indicate the vacuoles. Scale bars: 100 μm (E-F) and 20 μm (G). Data are presented as mean ± standard deviation; n = 6. *p<0.05, **p<0.01, ***p<0.001 compared with control.

**Description of additional supplementary files**

**Suppl.** **Video 1. Nutlin-3a induces the formation of huge vacuoles in *KRAS* MT/*p53* WT NSCLC cells**. The Z-stacking video of cells treated with DMOS or nutlin-3a (30 μM) for 24 h captured by holotomograpy microscope. Scale bar: 7 µm. The representative images are shown in Fig. 3B.

**Suppl. Video 2.** **Nutlin-3a induced vacuoles merge to form massive vacuoles with time and membrane rupturing in the end.** The time-lapse video was obtained by tracking nutlin-3a-treated H460 for 24 h at 17-min intervals using holotomograpy microscpe. Scale bar: 7 µm. The representative images are shown in Fig. 3C.

**Suppl. Video 3. GlcNAc rescues nutlin-3a-induced vacuoles in *KRAS* MT/*p53* WT NSCLC cells.** The Z-stacking video of cells treated with nutlin-3a (30 μM) with or without GlcNAc (40 mM) for 24 h captured by holotomograpy microscope. Scale bar: 7 µm. The representative images are shown in Fig. 6C.

**Suppl. Table 1. IC_50_ values of nutlin-3a in NSCLC and BEAS-2B cells.**

**Suppl. Table 2. List of genes upregulated exclusively in *KRAS* MT/*p53* WT NSCLC cells ^+^ compared to those in *KRAS* WT cells^++^.**

**^+^: A549, and H460**

**^++^: BEAS2B, and HCC827**

**Suppl. Table 3. List of genes downregulated exclusively in *KRAS* MT/*p53* WT NSCLC cells^+^ compare to those in *KRAS* WT cells^++^.**

**^+^: A549, and H460**

**^++^: BEAS2B, and HCC827**
